# Natural tristability of a confined helical filament with anisotropic bending rigidities

**DOI:** 10.1038/s41598-024-64903-x

**Published:** 2024-06-17

**Authors:** Zicong Zhou

**Affiliations:** Department of Physics, Tamkang University, No. 151 Yingzhuan Rd., Tamsui District, New Taipei City, 251301 Taiwan, ROC

**Keywords:** Metamaterial, Chiral multistable states, Confined material, Semiflexible biopolymer, Phase diagram, Biophysics, Materials science, Nanoscience and technology, Physics

## Abstract

We find that when $c_0 R\sim 0.5$ and $\tau _0R<0.11<c_0R$, confining a helical filament with anisotropic bending rigidities within a cylindrical tube of radius *R* can create a natural tristable status which is consisted of two low-pitch helices and one high-pitch helix, where a helical filament is referred to as a filament that has both an intrinsic curvature ($c_0$) and an intrinsic twist rate ($\tau _0$). The formation of the tristable status also requires that the filament has a significant difference between two bending rigidities and a large twisting rigidity. The relative heights of two low-pitch helices in a tristable status are close to zero, and the smaller the intrinsic twisting angle, the smaller the difference between these two heights. Moreover, at a large intrinsic twisting angle, two low-pitch helices display a large energy difference, and the energy difference increases with decreasing $\tau _0$. Meanwhile, the relative height of the high-pitch helix is always close to that of a straight line. Finally, at some specific intrinsic parameters, the tristable status can include an isoenergic status with two helices of the same energy but distinct pitches.

## Introduction

Metamaterials have attracted considerable attentions for decades owing to their fantastic properties and widely applications, such as for energy storage^1–5^, logical operation^6–8^, shape reconfigurable intelligent material^9–11^, electromagnetic material^12,13^, photonic material^14,15^, mechanical^4–7^ and thermal^16–18^ materials, etc. For instance, mechanical metamaterials can exhibit some unusual properties such as negative stiffness or negative compressibility^19,20^, negative Poisson’s ratio^21,22^, twisting under stretching or expanding under twisting^23,24^, ultralight^25,26^, ultra-stiff^27^, and ultra-strong with recoverability^28,29^. The extraordinary properties of metamaterials are based on multistability, i.e., a metamaterial can stay steadily in more than one stable or metastable states and switch among them. For instance, a stretching force or field can induce a switch of a multistable material from a stable or metastable state with a large size to a stable or metastable state with a small size, resulting in contraction or negative stiffness^19–22^. Similarly, such a material may also exhibit negative thermal expansion. However, in many cases a material requires external force or energy to maintain a deformed configuration, and such a requirement imposes limitation on its application. A typical example is that the twisted nematic liquid crystal requires an external field to maintain its parallel configuration^30,31^. A natural multistable material can overcome this problem since it can stay in several different stable or metastable configurations in absence of external forces so offers an ideal green energy material. However, identifying new natural multistable materials is a significant challenge.

On the other hand, confined materials often exhibit significantly different properties from their three-dimensional (3D) counterpart. A typical example is that a two-dimensional solar cell has usually a higher efficiency than that of the bulk one. Confinement may be even more crucial for biopolymers, as the cell is essentially a crowded system. For instance, MreB and its homologs appear in all cylindrical bacteria, and they are intrinsically straight, with a persistence length 5 to 10 times longer than the bacterial cell size^32,33^. However, within the cell, MreB can form either a helix or a ring and play crucial roles in many cellular functions, such as regulating cell shape, chromosome segregation, determining cell polarity, and organizing membranous organelles^33–35^. It was also reported that boundary constraint can result in unusual folding behaviors in responsive helical bilayer strips^36^.

Furthermore, helices and helical structures are ubiquitous and crucial owing to their valuable mechanical property or remarkable optical property^30,31,36–46^. The combination of helical and non-helical structures can also create some fantastic materials. For instance, the discovery of the alignment transition in nematic liquid crystal molecules from a parallel normally white mode to a twisted helical mode has led to the development of liquid crystal color displays^30,31^. Helix is also one of the simplest conformations of a filament. Therefore, the property of a helical filament in 3D space has been studied extensively^36,39–46^. Here a helical filament is referred to as a filament with both a finite intrinsic twisting rate (ITR) and a finite intrinsic curvature (IC)^39^, since the natural or force-free ground-state configuration (GSC) of its centerline is uniquely a helix^40^, and such a helix is also referred to as a free-standing helix. Microscopic filaments such as nanotubes and semiflexible biopolymers often possess finite IC and ITR^43–52^. For instance, a double-stranded DNA (dsDNA) has a finite ITR and special sequence order in a short dsDNA chain favors a finite IC^47–50^. Similarly, a long-range correlation in sequence can induce an IC for a dsDNA chain^51^. Moreover, helical configurations are metastable intermediates in the process of cholesterol crystallization in the native gallbladder bile^43–45,52^.

A natural question is then: what will happen if we confine a helical filament? It has been reported that confining a helical filament with isotropic bending rigidity inside a cylinder can create a natural bistable status which is consisted of two isoenergic stable helices or one stable helix and the other a metastable helix^53,54^. Here, isotropic bending rigidity means that two bending rigidities associated with the two principal axes inertia of the crosssection of the filament are the same, as seen in a uniform filament with a circular or square crosssection. However, a filament is not necessary required to have isotropic bending rigidities, even if it is uniform, as it may have a non-circular or non-square crosssection. For instance, many fibers are flat. Another example is that a dsDNA has a non-circular crosssection since it is consisted of two nucleotide bases connected by hydrogen bonds^41,44^. It then raises another intrigue question: would anisotropy in bending rigidities strengthen or weaken the bistablility of a confined helical filament? In this work, we report that anisotropy can induce a split of the low-pitch helix, resulting in a natural tristable state. This state consists of two low-pitch helices and one high-pitch helix, with these helices having either similar energies or significantly different energies.

The outline of the paper is as follows. “Model and methods" section first outlines the elastic model employed for the filament in this study. Subsequently, it utilizes standard variational techniques and stability analysis method to derive static equations and stability criteria for a helix. “Triple stability" section presents the findings of this paper. The work is concluded with a summary and some remarks in “Discussions" section.

## Model and methods

### Model

Denoting the arclength of its centerline as *s* and the locus of centerline as **r**(*s*), the conformation of a filament can be described by a triad of unit vectors $\{\textbf{t}_i(s)\}_{i=1,2,3}$, where **t**$_1$ and **t**$_2$ are oriented along the principal axes of the crosssection, $\textbf{t}_3\equiv \dot{\textbf{r}}=\textbf{t}_1\times \textbf{t}_2$ is the unit tangent to the centerline^40,45,55^ and the symbol “ $\dot{\phantom{a}}$ " represents the derivative with respect to *s*. The relation among $\textbf{t}_i(s)$’s is given by the generalized Frenet equations $\dot{\textbf{t}}_i={\varvec{\omega }}\times \textbf{t}_i$^40,45,55^, where ${\varvec{\omega }}=(\omega _1, \omega _2, \omega _3)$ represents curvature and torsion parameters. Furthermore, we can use Euler angles $\theta$, $\phi$ and $\psi$ to represent $\textbf{t}_i$ and ${\varvec{\omega }}$, as^40,45,55,56^,

1 $$\begin{aligned} \textbf{t}_3= & {} (\sin \phi \sin \theta ,-\cos \phi \sin \theta ,\cos \theta ), \end{aligned}$$

2 $$\begin{aligned} \omega _1= & {} \sin \theta \sin \psi \ \dot{\phi }+\cos \psi \ \dot{\theta }, \ \omega _2=\sin \theta \cos \psi \ \dot{\phi }-\sin \psi \ \dot{\theta }, \ \omega _3=\cos \theta \ \dot{\phi }+\dot{\psi }. \end{aligned}$$

The energy of a uniform filament can be written as^41,45,55^

3 $$\begin{aligned} E=\int _0^L\varepsilon ds, \ \varepsilon ={k_1\over 2}(\omega _1-\omega _{10})^2 +{k_2\over 2}(\omega _2-\omega _{20})^2+{k_3\over 2}\left( \omega _3-\tau _0\right) ^2, \end{aligned}$$

where $k_1$ and $k_2$ are bending rigidities associated with two principal axes inertia of crosssection, $k_3$ is twisting rigidity, $\omega _{10}$ and $\omega _{20}$ are components of IC and the magnitude of IC is $c_0=\sqrt{\omega _{10}^2+\omega _{20}^2}$, $\tau _0$ is ITR. *L* is the total contour length and is a constant, i.e., we consider an inextensible filament. We also let $\omega _{10}=c_0\sin \alpha$ and $\omega _{20}=c_0\cos \alpha$, where $\alpha$ represents an intrinsic twisting angle of the cross-section around the centerline^57^. When $k_2=k_1$, i.e., for a filament with isotropic bending rigidities, $\alpha$ appears as a constant added to $\psi$ or in the form of $\psi -\alpha$ so can be ignored^53,54^. However, in anisotropic case $k_2\ne k_1$, there is no way to neglect $\alpha$ so we have to deal with six intrinsic parameters, i.e., $c_0$, $\tau _0$, $k_1$, $k_2$, $k_3$ and $v_0$, two more than those in an isotropic system. A free-standing helix has $\cos \theta =\tau _0/\sqrt{c_0^2+\tau _0^2}$, radius $R_h^0=c_0/(c_0^2+\tau _0^2)\le 1/c_0$, pitch=$2\pi \tau _0/(c_0^2+\tau _0^2)$ and $\varepsilon =0$, regardless of $k_i$^40,45^. The pitch of a helix is the height of one complete helix turn measured along the axis of helix, and a small $\tau _0$ yields a low-pitch free-standing helix.

Note that $k_i$’s are different from stiffness or elastic moduli $\kappa _i$, but $k_1=\kappa _1I_1$, $k_2=\kappa _2I_2$, and $k_3=\kappa _3(I_1+I_2)$, where $I_i$ are the principal moments of inertia and are dependent on the shape of the crosssection^37,38^. $\kappa _1$ and $\kappa_2$ are also called Young’s modulus, and $\kappa _3$ is usually called shear modulus^37,38^. Consequently, in elastic theory an isotropic filament is usually referred to as a filament with $\kappa _1=\kappa _2$, but in this case $k_1$ can be different from $k_2$ if $I_1\ne I_2$. In other words, even for an isotropic filament, we can still adjust the ratio of $k_2/k_1$ by changing the shape of the crosssection. For convenience, henceforth we call the system with isotropic bending rigidities as an isotropic system, and similarly for the anisotropic system.

This model is often used to model a semiflexible biopolymer such as DNA, RNA and proteins. For instance, for a dsDNA at temperature $T=298K$, $k_1/k_BT\approx 50$ nm, $k_3/k_BT\approx 75$ nm where $k_B$ is the Boltzmann constant, and $\tau _0\approx 1.76$ nm$^{-1}$^58,59^.

When $c_0<1/R$ clearly and $k_i\ne 0$, the confinement is very strong, where *R* is the radius of a cylindrical tube used to confine the filament. Consequently, to reduce bending energy effectively, the filament tends to touch the wall of the tube, or it is in fact confined on the surface of the tube. It then results in a constraint on $\textbf{r}$ so $x=R(1-\cos \phi )$, $y=-R\sin \phi$ and $\dot{\phi }=\sin \theta /R$^46^. When $k_1=k_2$, the confined system has been well studied^53,54^. For simplicity, henceforth we also scale *L* by *R* and *E* by $k_1/R$, i.e., let $R=1$ and $k_1=1$.

### Static equations

We can find stable configurations of the system by minimizing *E* and it results in the following two static equations:

4 $$\begin{aligned} {\partial \varepsilon \over \partial \theta } -{d\over ds}{\partial \varepsilon \over \partial \dot{\theta }}={\partial \varepsilon \over \partial \psi } -{d\over ds}{\partial \varepsilon \over \partial \dot{\psi }}=0. \end{aligned}$$

Explicit forms of static equations are lengthy so we present them in “Appendix” as Eqs. (9)-(10). They are second order nonlinear differential equations so that there is no way to find their general solutions. However, it is straightforward to obtain helical solution since owing to symmetry, to have a helical configuration for the filament implies to take $\theta =\theta _h$ as a *s*-independent constant. The existence of other stable or metastable solutions of the static equations, aside from the helix, remains an unsolved problem. However, it should be noted that Monte Carlo simulations for the discretized isotropic system suggest that the helix is the unique steady state within the range of interesting parameters, even for a short chain^53^, and we can expect the same result for an anisotropic system. Without lose of generality, we let $\pi /2\ge \theta _H\ge 0$, and define the relative extension or height as $z_r\equiv z(L)/L$ so $z_r=\cos \theta _h$ for a helix. It follows that for a free-standing helix $z_r\approx \tau _0/c_0<<1$ when $\tau _0<<c_0$ so it looks like a circle. Let $\theta =\theta _h$, $\psi =\psi _h$ which is also a constant, $v=\cos \psi _h$ and $v_0=\cos \alpha$, static equations and $\varepsilon$ are reduced into

5 $$\begin{aligned}{} & {} [k_3+2(\pm c_0\sqrt{1-v^2}\sqrt{1-v_0^2}-1+v((1-k_2)v+k_2c_0v_0)]z_r\sqrt{1-z_r^2}+k_3\tau _0(2z_r^2-1)\nonumber \\- & {} 2[k_3-1+(1-k_2)v^2]z_r^3\sqrt{1-z_r^2}=0,\end{aligned}$$

6 $$\begin{aligned}{} & {} [(k_2-1)(1-z_r^2)v -c_0k_2v_0]\sqrt{1-v^2}\pm c_0v\sqrt{1-v_0^2}=0,\end{aligned}$$

7 $$\begin{aligned} \varepsilon= & {} {1\over 2}\left[ 1+c_0^2(1+(k_2-1)v_0^2)+(k_3-2-(k_3-1)z_r^2)z_r^2-2c_0 (k_2vv_0 \pm \sqrt{1-v^2}\sqrt{1-v_0^2})(1-z_r^2)\right. \nonumber \\{} & {} \left. +(k_2-1)v^2(1-z_r^2)^2-2k_3z_r\sqrt{1-z_r^2}\tau _0+ k_3\tau _0^2\right] , \end{aligned}$$

where the sign $``\pm "$ comes from $\sin \psi =\pm \sqrt{1-v^2}$ and $\sin \alpha =\pm \sqrt{1-v_0^2}$ so that in Eqs. (5)-(6), it takes +(-) if $\sin \psi$ has the same (different) sign as that of $\sin \alpha$.

### Stability criterion

Equations (5) and (6) have clearly multiple solutions for both $z_r$ and *v*. But even for a real solution, the filament can still be unstable since it can correspond to either a maximum or a saddle point in *E*. To examine stability of a helix, similar to that in Refs.^53,54^, we firstly linearize static equations by setting $\theta =\theta _h+\Delta \theta$, $\psi =\psi _h+\Delta \psi$ and keep the terms up to the first order to obtain two linear constant coefficients differential equations for $\Delta \theta$ and $\Delta \psi$, and then we assume $\Delta \theta =B_\theta e^{\gamma s+\delta }$ and $\Delta \psi =B_\psi e^{\gamma s+\delta }$, and demand novanishing $B_\theta$ and $B_\psi$ so obtain a quadratic equation for $\gamma ^2$, i.e.,

8 $$\begin{aligned} \Delta\equiv & {} k_3[k_2+(1-k_2)v^2]\gamma ^4+C_2\gamma ^2+C_0=0, \end{aligned}$$

where $C_0$ and $C_2$ are independent of *s* and $\gamma$. If both $\gamma ^2<0$, then the helix is at least metastable; otherwise, the helix is unstable. In other words, $\gamma ^2$ can be used as stability criterion of a helix. To determine GSC we need to compare $\varepsilon$ further even both $\gamma ^2<0$ since it may exist multiple stable or metastable configurations. The linearized static equations and detail expressions of $C_0$ and $C_2$ are also presented in “Appendix”.

We should also stress that owing to strong nonlinearity of the static equations, when $\gamma ^2\sim 0$, $\varepsilon$ can be still in maximum even both $\gamma ^2<0$ because of the effect of higher order terms in expansions of Eqs. (9) and (10). Therefore, we also examine $\partial ^2\varepsilon /\partial z_r^2$ and $\partial ^2\varepsilon /\partial v^2$ to exclude these improper results.

## Triple stability

Our resultss are based on solving Eqs. (5), (6) and (8) exactly. For a given set of parameters ($c_0$, $\tau _0$, $k_1$, $k_2$, $k_3$ and $v_0$), we first solve Eqs. (5) and (6) to find the relevant values of *v* and $z_r$, and then substitute these values into Eq. (8) to find $\gamma ^2$.

### When $c_0=0.5$

Since the isoenergic bistable state for the isotropic system occurs at $c_0=0.5$^53,54^, in this work we also start from $c_0=0.5$. We do not consider $k_2>10$ and $k_3>10$ since it should be unpractical. We also find that there is no longer tristable helix when $\tau _0>0.11$ though bistable helix is still possible in either anisotropic or isotropic system.

**Figure 1:**
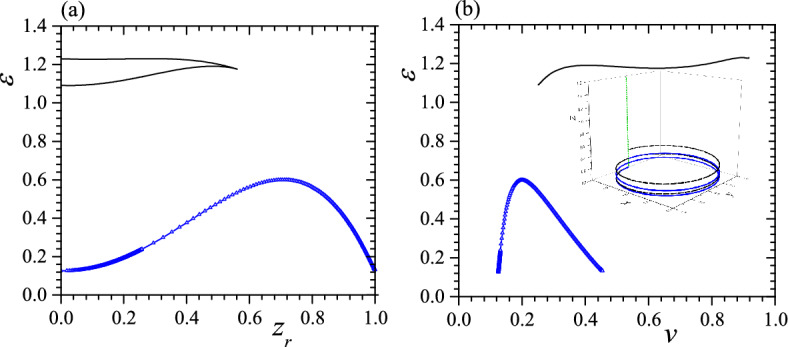
(**a**) $\varepsilon$ vs. $z_r$ when $c_0=0.5$, $\tau _0=0.01$, $k_2=2.5$, $k_3=5$ and $v_0=0.2$, (**b**) $\varepsilon$ vs. *v* with the same parameters as in (**a**). The blue lines with empty triangle and the black lines are obtained by taking “+" and “−" in Eqs. (5)–(7), respectively. The inset in (**b**) presents three helices with $z_r=0.0125$ (solid blue with 2 turns), $z_r=0.0252$ (black dashed with 2 turns) and $z_r=0.999918$ (green with ball and 1/4 turn). The corresponding free-standing helix has $z_r=0.0200$. Reduced units are used.

The typical relationships between $\varepsilon$, $z_r$ and *v* when $c_0=0.5$, $\tau _0=0.01$, $k_2=2.5$, $k_3=5$ and $v_0=0.2$ are presented in Fig. 1 in which the blue lines with empty triangle and the black lines are obtained by taking “+" and “−" in Eqs. (5)–(7), respectively.

**Figure 2:**
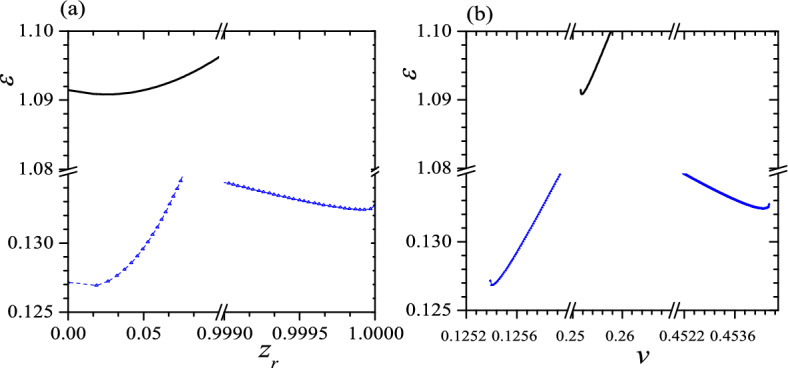
Enlargement of neighborhood of minima in Fig. 1. The symbols are the same as that in Fig. 1. Reduced units are used.

It is not difficult to identify maxima of $\varepsilon$ in Fig. 1, but the minima of $\varepsilon$ is unclear because they are in two edges of curves so in Fig. 2 we enlarge neighborhoods of minima in Fig. 1. From the blue lines in Figs. 1 and 2, we can know that the result obtained from $\varepsilon$ vs. $z_r$ curve agrees with that obtained from $\varepsilon$ vs. *v* curve, i.e., both curves show two minima and one maximum in $\varepsilon$ and two minima occur at (1) $v=0.1254$, $z_r=0.0125$ and $\varepsilon =0.1269$; (2) $v=0.4544$, $z_r=0.999918$ and $\varepsilon =0.1324$. It gives two stable or metastable helices with significantly different pitches but only a small difference in $\varepsilon$. In contrast, in black lines of Figs. 1a and 2a, we find one minimum and one maximum in $\varepsilon$. The minimum $\varepsilon$ occurs at $v=0.2518$, $z_r=0.0252$ and $\varepsilon =1.0908$ so is different that obtained from the blue lines. However, in black lines of Figs. 1b and 2b, we find two minima and two maxima in $\varepsilon$ or there is one more minimum in $\varepsilon$ vs. *v* curve than that in $\varepsilon$ vs. $z_r$ curve, so clearly this extra minimum in $\varepsilon$ corresponds to a saddle point or a unstable status. In other words, the black lines offer only a low-pitch metastable helix and the metastability is due to a rather high $\varepsilon$ in this status. The calculation of $\gamma ^2$ confirms above conclusion, i.e., with this set of parameters the system exhibits a tristable status which is consisted of three stable or metastable helices. Two of three helices have very low pitches ($z_r\sim 0$), resembling a circle, while the third has a rather high-pitch ($z_r\sim 1$), resembling a straight line. Two low-pitch helices also have considerably different $\varepsilon$. We also find that the helix with the smallest $z_r$ always exhibits the lowest $\varepsilon$, making it the GSC of the system. The two higher-pitched helices are then identified as metastable states. Additionally, in this case, the free-standing helix has $z_r=\tau _0/\sqrt{c_0^2+\tau _0^2}=0.0200$, positioning it between the $z_r$s of two confined low-pitch helices. This suggests that the free-standing helix undergoes a split into two low-pitch helices due to anisotropy.

**Figure 3:**
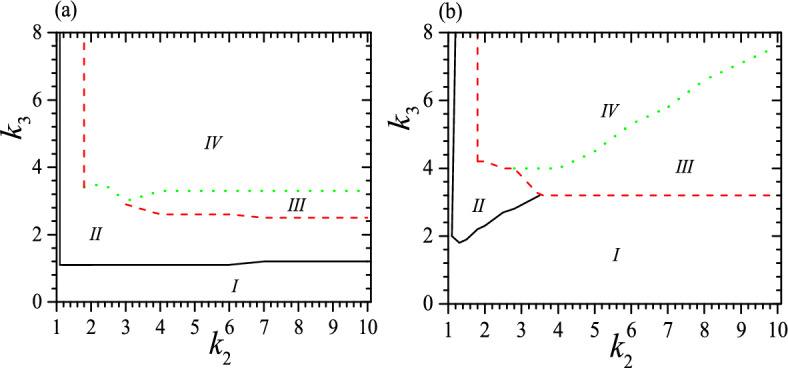
Phase diagrams for the system at $c_0=0.5$ and (**a**) $\tau _0=0.01$; (**b**) $\tau _0=0.05$. In (**a**), regime I has not stable helix or has only one stable low-pitch helix and is bound by black solid line, $k_2$ and $k_3-$axis; regime II has two stable or metastable helices with quite different pitches and is enclosed by black solid line, red dashed and green dotted lines; regime III has two stable or metastable low-pitch helices and is enclosed by red dashed and green dotted lines; regime IV is the tristable regime and is bound by red dashed and green dotted lines. In (**b**), four regimes have the same meaning as that in (**a**), but regime *I* is bound by black solid line, $k_2$, $k_3$-axis and red dashed line; regime II is enclosed by black solid and red dashed line; regime III is enclosed by red dashed and green dotted lines again; regime *IV* is also bound by red dashed and green dotted lines. Reduced units are used.

The phase diagrams for the system can be divided into four regimes, as shown in Fig. 3 when $c_0=0.5$, $\tau _0=0.01$ and 0.05. In regime I a helix is unstable or there is only one stable low-pitch helix; regime II has two stable or metastable helices with quite different pitches and it is similar to that of the isotropic system^53,54^; in regime III there are two stable or metastable low-pitch helices and is clearly different from that in the isotropic system^53,54^; in regime IV we can find three stable or metastable helices. The tristable status appears when $c_0\sim 0.5$, $\tau _0<<1$, $k_2>1$ and $k_3>1$ clearly, and in a proper range of $v_0$. For instance, when $\tau _0=0.01$, there is not tristable status when $k_2<1.8$ and at $k_2=1.8$, and tristable status requires both $k_3>3.4$ and $0.12>v_0>0$. From Fig. 3, we can see that the boundary of different regimes is strongly dependent on $\tau _0$. At a small $\tau _0$, the threshold of $k_3$ for regime II is almost flat with the variation of $k_2$, but at a large $\tau _0$ it increases fast up to a moderate $k_2$ and has no longer bistable status at a large $k_2$. Meanwhile, the threshold of $k_3$ for regime III decreases slowly with increasing $k_2$ at a small $\tau _0$, but at a large $\tau _0$ it decreases fast with increasing $k_2$ up to a moderate $k_2$ and then becomes flat at a large $k_2$. Moreover, the threshold of $k_3$ for regime IV is almost flat at a small $\tau _0$, but increases almost linear at a larger $\tau _0$. In general,the larger the $\tau _0$, the larger the required $k_3$ for regimes III and IV as well as the larger the area of regimes I and III. In contrast, the area of regime II shrinks with increasing $\tau _0$.

To obtain phase diagrams, for a given set of $c_0$ and $\tau _0$, we vary $k_2$ and $k_3$ to solve Eqs. (5), (6) to find the corresponding values of *v* and $z_r$. We then substitute these values into Eq. (8) to find relevant $\gamma ^2$, in order to determine whether these parameters offer a stable or metastable state. The lines or boundaries of different regimes in the phase diagrams are determined by $|\gamma ^2|\le 0.1^{-5}$.

The regime *I* is trivial since it is rather easy to obtain the same helix or non-helix in free space, though the parameters are in general different. Regime II is similar to the bistable regime in an isotropic system^53^, and practically it is much easier to obtain isoenergic bistable status in an isotropic system so that the regime II is less significant. In contrast, regimes III and IV are brand new and denote considerable effect of anisotropy. These two regimes possess a low-pitch but high energy metastable configuration which is absented in an isotropic system^53^. Due to its increased flexibility, a material with a tristable state should have a broader range of applications than one with a bistable state.

**Figure 4:**
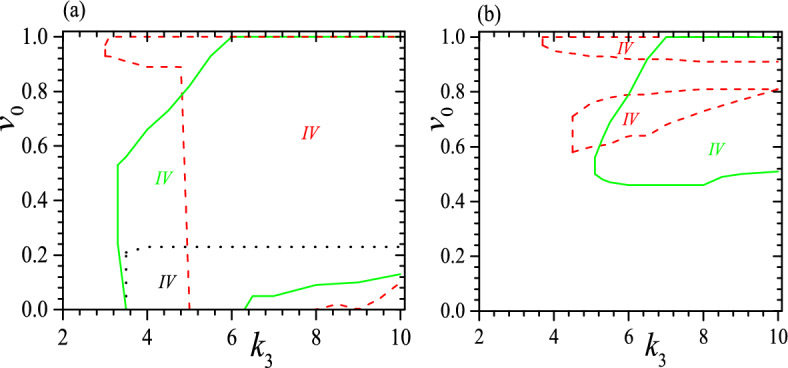
Tristable regime (IV) when $c_0=0.5$ and (**a**) $\tau _0=0.01$, $k_2=2$ (black dotted), 3 (red dashed) and 6 (green solid), (**b**) $\tau _0=0.05$, $k_2=3$ (red dashed) and 6 (green solid). The black dotted line and $k_3$-axis enclose tristable regime for $k_2=2$. The red dashed line and $k_3$-axis enclose tristable regime for $k_2=3$. The green solid line and $k_3$-axis enclose tristable regime for $k_2=6$. Different colors of IV correspond to different parameters. Reduced units are used.

We should note that Fig. 3 illustrates only the necessary conditions for the formation of a tristable status, as it does not take into account the effect of $v_0$. A complete picture on the stability is also dependent on $v_0$. For instance, when $\tau _0=0.01$ and $k_2=2$, to have a tristable status requires $0.23\ge v_0>0$ when $k_3\ge 4$. Some typical examples for the effect of $v_0$ are presented in Fig. 4 for $c_0=0.5$, $\tau _0=0.01$, $k_2=2$, 3 and 6, as well as $c_0=0.5$, $\tau _0=0.05$, $k_2=3$ and 6. We find again that a small $\tau _0$ favors tristable status, so it appears when $\tau _0=0.01$ and $k_2=2$ but vanishes when $\tau _0=0.05$ and $k_2=2$. Meanwhile, the range of $v_0$ for a tristable status shrinks obviously at a large $\tau _0$, as we can see from a comparison between Fig. 4 a and b. Moreover, at a small $\tau _0$ and up to a moderate $k_2$, to have a tristable status requires $v_0^1>v_0>0$, as we can see from the black dotted lines in Fig. 4a. $v_0^1$ is almost independent of $k_3$ but it increases with increasing $k_2$, and $v_0^1$ can reach a maximum which is dependent on both $\tau _0$ and $k_2$. For instance, when $\tau _0=0.01$, the maximum occurs at $k_2=2.6$ with $v_0^1=0.57$. However, when $k_2$ is rather large ($k_2>2.7$ when $\tau _0=0.01$), the range of $v_0$ for a tristable status does not start at $v_0=0$, but can begin from a rather large $v_0$, as we can see from the beginning of red dashed and green solid lines in Fig. 4. Furthermore, at a moderate $k_2$ the range of $v_0$ for a tristable status can reach the maximum, i.e., $1>v_0^1>0$, and this should be useful because to realize a required $v_0$ or $\alpha$ in practical application may be uneasy. Larger $k_3$ does not always favor a larger range of $v_0$, as we can see from the red dashed and green solid lines in Fig. 4. Our calculations show further that at a moderate $\tau _0$ and $k_2$, the tristable regime can be divided into two pieces and between them the helix with a middle pitch is unstable, shown as regimes enclosed by red dashed lines in Fig. 4b. We find further that when $\tau _0\ge 0.03$, there is no longer a full range of $v_0$ for tristable helix, as shown in Fig. 4b. In a word, the range of $v_0$ for tristable regime is neither a simple function of $k_2$ nor $k_3$.

**Figure 5:**
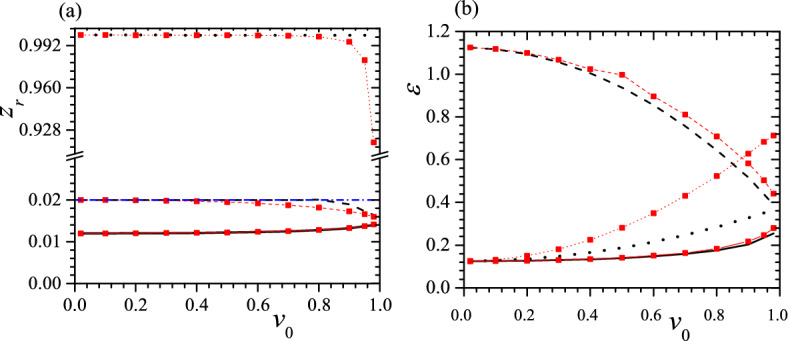
$z_r$ (**a**) and $\varepsilon$ (**b**) vs. $v_0$ for tristable status at $c_0=0.5$, $\tau _0=0.01$, $k_2=3$, $k_3=6$ (black solid, dashed and dotted lines) and $k_2=k_3=6$ (red solid, dashed and dotted lines with red square). Blue dash-dotted line in (**a**) represents $z_r$ of a free-standing helix. In both figures, solid lines give the helix with the lowest pitch, dashed lines are correspond to the helix with the middle pitch and dotted lines present the helix with the highest pitch. Reduced units are used.

**Figure 6:**
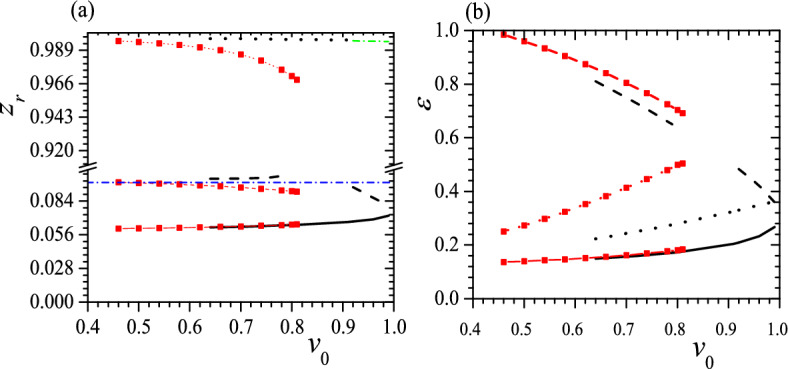
$z_r$ (**a**) and $\varepsilon$ (**b**) vs. $v_0$ for tristable status at $c_0=0.5$, $\tau _0=0.05$, $k_2=3$, $k_3=6$ (black solid, dashed and dotted lines) and $k_2=k_3=6$ (red solid, dashed and dotted lines with red square). The meaning of symbols is the same as that in Fig. 5. Reduced units are used.

The differences between $z_r$ and $\varepsilon$ of three helices in a tristable status are also crucial, so in Figs. 5 and 6 we present some typical relations between $z_r$, $\varepsilon$ and $v_0$ when $c_0=0.5$, $\tau _0=0.01$, 0.05, $k_2=3$, $k_3=6$ and $k_2=k_3=6$. From these figures, at first, we can see that $z_r\sim 0$ for both low-pitch helices, and the larger the $v_0$, the closer the two $z_r$s, as shown in Figs. 5a and 6a for black solid, black dashed and red solid, red dashed lines. Meanwhile, up to a moderate $v_0$, two low-pitch helices have a considerable difference in $\varepsilon$, and the smaller the $\tau _0$, the larger the difference in $\varepsilon$, as shown in Figs. 5b and 6b. Second, $z_r\sim 1$ for the highest pitch helix and the smaller the $\tau _0$, the closer the $z_r$ to 1, so the highest pitch helix is almost indistinguishable from a straight line, as shown in Figs. 5a and 6a. Third, at a small $\tau _0$, a large $k_2$, $k_3$ and $v_0$, two metastable helices can have the same $\varepsilon$ or in an isoenergic state, shown as the crossovers of two red lines in Fig. 5b and two black lines in Fig. 6b. Fourth, similar to Fig. 4b, at a moderate $\tau _0$ and $k_2$, the tristable regime can be divided into two pieces and between them the helix with a middle pitch is unstable, shown as black dashed lines in Fig. 6. Finally, we find that $z_r$ of a free-standing helix is closer to that of the helix with a middle pitch, as shown in Figs. 5a and 6a.

Together with the result from the isotropic system^53^, we can figure out the mechanism behind the formation of the tristable state. It arises from the interplay and interdependence of bending, twisting, confinement, and anisotropy. In free space, the natural shape of the filament is a low-pitch helix. Strong confinement favors a straight filament, resulting in a high-pitch helix and leading to a bistable state in the isotropic system. Furthermore, anisotropy is an intrinsic property that tends to alter the inherent configuration, causing a split of the free-standing helix and giving rise to a tristable state.

### When $c_0=0.45$ and 0.55

**Figure 7:**
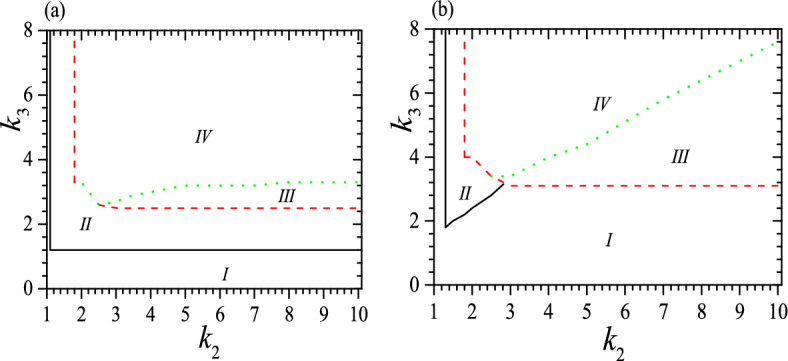
Phase diagrams for the system at $c_0=0.45$ and (**a**) $\tau _0=0.01$, (**b**) $\tau _0=0.05$. The meaning of symbols is the same as that in Fig. 3. Reduced units are used.

**Figure 8:**
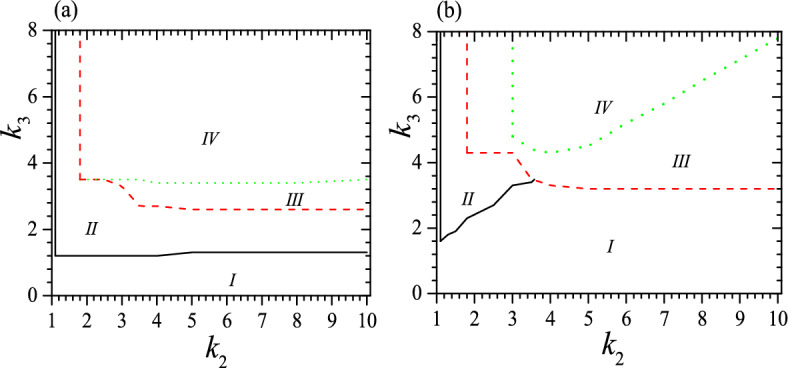
Phase diagrams for the system at $c_0=0.55$ and (**a**) $\tau _0=0.01$, (**b**) $\tau _0=0.05$. The meaning of symbols is the same as that in Fig. 3. Reduced units are used.

**Figure 9:**
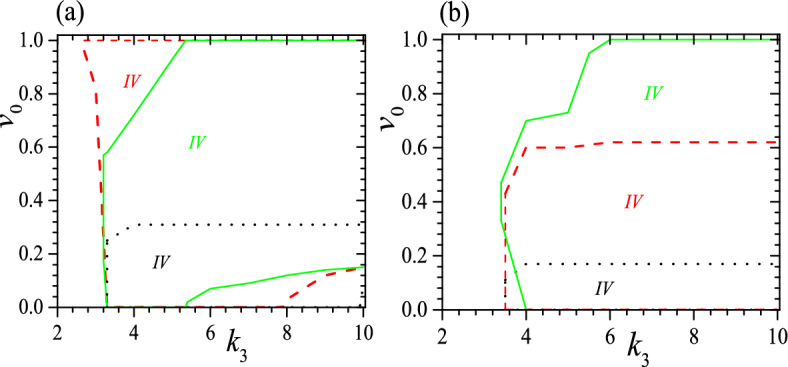
Tristable regimes (IV) when $c_0=0.45$ (**a**), 0.55 (**b**), $\tau _0=0.01$, $k_2=2$ (black dotted), 3 (red dashed) and 6 (green solid). The black dotted line and $k_3$-axis enclose the tristable regime for $k_2=2$. The red dashed line, $k_3$-axis and $v_0=1$ enclose the tristable regime for $k_2=3$. The green solid line, $k_3$-axis and $v_0=1$ enclose the tristable regime for $k_2=6$. Different colors of *IV* correspond to different parameters. Reduced units are used.

Next we explore the influence of $c_0$. Phase diagrams for tristable state at $c_0=0.45$, 0.55, $\tau _0=0.01$ and 0.05 are shown in Figs. 7 and 8. Comparing Figs. 7 and 8 with Fig. 3, we can know that the basic characters are similar for three $c_0$s. In particular, they all have four regimes in phase diagrams and have similar dependence of four regimes on $k_2$, $k_3$ and $\tau _0$. It is also clear that a smaller $c_0$ favors the tristable status, as these figures show that it reduces the required $k_3$ effectively. This fact is confirmed further by examining the dependence of $v_0$, as shown in Fig. 9 for the system with $c_0=0.45$, 0.55, $\tau _0=0.01$, $k_2=2$, 3 and 6. Comparing Fig. 9 with Fig. 4a, we can find that a smaller $c_0$ enlarges considerably the range of $v_0$ for tristable status. For instance, compared to that at $c_0=0.5$, the area enclosed by red dashed lines increases significantly when $c_0=0.45$ but shrinks clearly when $c_0=0.55$. This is a natural result since the strong confinement favors the formation of two higher pitch helices or favors the tristable status.

**Figure 10:**
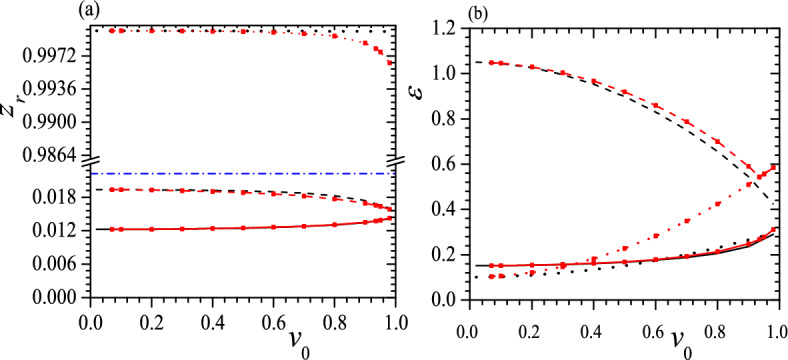
$z_r$ (**a**) and $\varepsilon$ (**b**) vs. $v_0$ for tristable status at $c_0=0.45$, $\tau _0=0.01$, $k_2=3$, $k_3=6$ (black solid, dotted, dashed and dotted lines) and $k_2=k_3=6$ (red solid, dashed and dotted lines with red square). The meaning of symbols is the same as that in Fig. 5. Reduced units are used.

**Figure 11:**
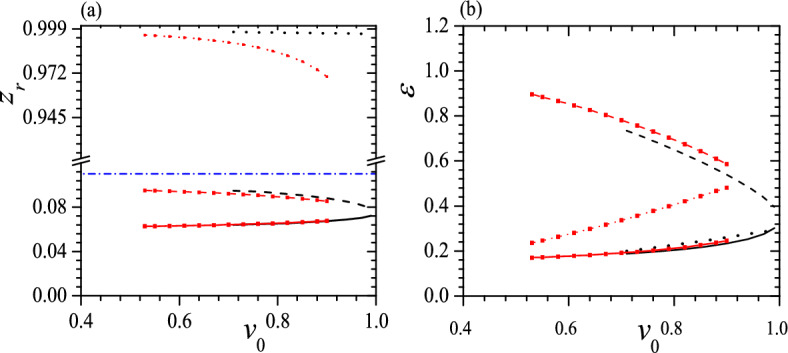
$z_r$ (**a**) and $\varepsilon$ (**b**) vs. $v_0$ for tristable status at $c_0=0.45$, $\tau _0=0.05$, $k_2=3$, $k_3=6$ (black solid, dashed and dotted lines) and $k_2=k_3=6$ (red solid, dashed and dotted lines with red square). The meaning of symbols is the same as that in Fig. 5. Reduced units are used.

**Figure 12:**
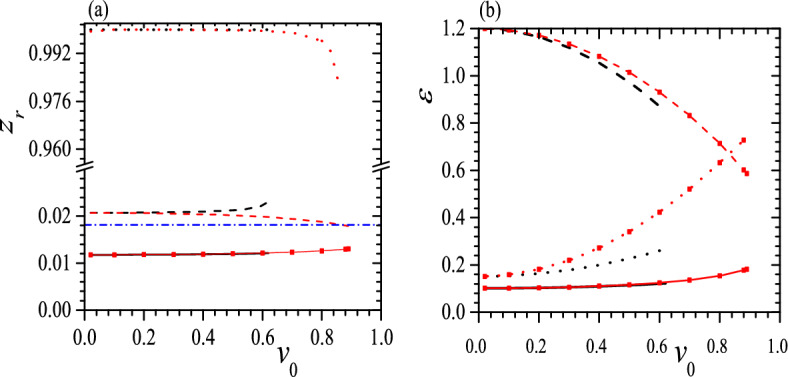
$z_r$ (**a**) and $\varepsilon$ (**b**) vs. $v_0$ for tristable status at $c_0=0.55$, $\tau _0=0.01$, $k_2=3$, $k_3=6$ (black solid) and $k_2=k_3=6$ (red dashed line with red square). The meaning of symbols is the same as that in Fig. 5. Reduced units are used.

**Figure 13:**
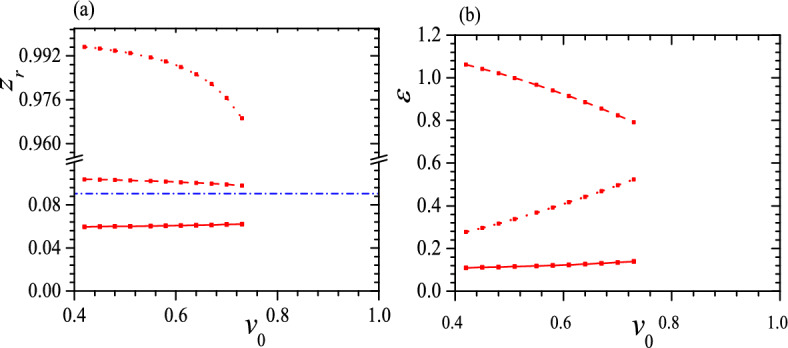
$z_r$ (**a**) and $\varepsilon$ (**b**) vs. $v_0$ for tristable status at $c_0=0.55$, $\tau _0=0.05$ and $k_2=k_3=6$ (red dashed line with red square). The meaning of symbols is the same as that in Fig. 5. Reduced units are used.

Figures 10, 11, 12 and 13 exhibit some typical relations between $z_r$, $\varepsilon$ and $v_0$ for tristable status when $c_0=0.45$, 0.55, $\tau _0=0.01$, 0.05, $k_2=3$, $k_3=6$ and $k_2=k_3=6$. When $c_0=0.55$, $\tau _0=0.05$, $k_2=3$ and $k_3=6$, the tristable regime is too narrow to illustrate so it disappears in Fig. 13. Comparing Figs. 12 and 13 with Figs. 5 and 6, we can find that different $c_0$ result essentially the same features, except for two special cases. The first significant difference is that when $c_0<0.5$, the lowest pitch helix is not always the GSC, as is the case for $c_0=0.5$ and 0.55; instead, the highest pitch helix can become the GSC owing to the stronger confinement, shown as black lines in Figs. 10b and 11b. The second notable difference is that the tristable regime consists of only one piece at $c_0>0.5$ because weak confinement disfavors the formation of high-pitch helix, as shown in Fig. 13. Moreover, at some special $v_0$ the GSC becomes an isoenergic state besides a low-pitch metastable helix, or the ground state has two stable helices with the same $\varepsilon$ but quite different $z_r$, and the smaller the $\tau _0$, the smaller the $v_0$ for the isoenergic status, shown as the crossover of black solid line and black dotted line in Figs. 10b and 11b. Again, $z_r$ of a free-standing helix is closer to that of the helix with the middle pitch, as shown in Figs. 10a, 11, 12 and 13a.

## Discussions

In summary, based on the elastic theory we find a mechanism to form a natural tristable system. Our results demonstrate that when $c_0 R\sim 0.5$ and $\tau _0R<0.11<c_0R$, confining a helical filament with anisotropic bending rigidities within a cylinder can create a natural tristable state. The tristable state arises from the competition and cooperation among bending, twisting, confinement, and anisotropy. In free space, the natural shape of such a filament is a low-pitch helix. Strong confinement tends to straighten the filament, resulting in a stable high-pitch helix and leading to a bistable state if the system is isotropic. Furthermore, anisotropy induces the split of the low-pitch free-standing helix, ultimately resulting in a tristable state. We anticipate that this mechanism also works in other confinements, such as square or rectangle tubes, even though the stable configuration of the filament may no longer be a helix.

We find that the smaller the $\tau _0$, the easier to realize the tristable status. The tristable status is consisted of two low-pitch helices and one high-pitch helix, and these helices can have either nearly the same energy or significantly different energy. The formation of the tristable state also requires a large twisting rigidity and a substantial disparity between two bending rigidities, i.e., a strong anisotropy. The phase diagrams of this system can be divided into four regimes or the system has four distinct statuses. In regime I a helix is unstable or there exists only one stable low-pitch helix; in regime II there are two stable or metastable helices and these two helices have notably different pitches; in regime III there are two stable or metastable low-pitch helices; in regime IV there exists three stable or metastable helices. Regimes III and IV are brand new and exhibit the effect of anisotropy. They possess a low-pitch but high energy metastable configuration which is absented in an isotropic system^53^. We find that the relative heights of the two low-pitch helices are close to zero, making them resemble a circle, and the larger the $v_0$, the smaller the difference between two heights. Moreover, up to a moderate $v_0$, two low-pitch helices have a rather large difference in energy, and the smaller the $\tau _0$, the larger the difference. Meanwhile, the height of the high-pitch helix is almost indistinguishable from a straight line. We also find that at a small $\tau _0$, a large $k_2$, $k_3$ and $v_0$, two higher pitch helices can possess the same $\varepsilon$ or the filament can be in a metastable isoenergic state. Finally, at some special $v_0$ the GSC of the system is in an isoenergic state, i.e., the tristable status has two stable helices with the same $\varepsilon$ but significant different $z_r$, in addition to a low-pitch metastable helix.

While our results are based on the elastic model and may appear to have limited relation to real materials, they still provide valuable insights for identifying relevant materials. From our results, intuitionally, to have a tristable state, in free space the free-standing helix should exhibit two distinct visible characters: (1) a non-circular or non-square crosssection, resulting in a large $k_2/k_1$; (2) a low-pitch, leading to a small $\tau _0$. Many nanotubes or proteins, such as actin and actin complexes, exhibit both of these two characteristics. In accord with these two characters, we can first rule out MreB and its homologs since they are intrinsically straight. Additionally, a tristable system requires $\tau _0/c_0<0.11/0.5=0.22$. It was reported that tandem sequence repeats of approximately 126 adenine tracts in dsDNA can yield a circle structure^48,49^. This gives rise to $c_0=2\pi /(126*0.34)=0.147$nm$^{-1}$, which is much smaller than $\tau _0=1.76$nm$^{-1}$. As a result, constructing a tristable system using dsDNA is impractical unless some bases can be removed to achieve a smaller $\tau _0$ and a larger $c_0$.

Finally, our findings are valid to both macroscopic and microscopic systems, but the microscopic version, i.e., a nano-filament under nanoconfinement, should be more significant. Due to its increased flexibility, a material exhibiting natural tristable behavior would have a broader range of applications than one with bistable characteristics, making this new system particularly intriguing. This natural tristable system may also offer a prospective green metamaterial since it does not need to consume energy to maintain one of its three stable or metastable configurations. Owing to the inherent chiral symmetry of a helix, this tristable system may exhibit remarkable optical properties, potentially becoming optically active materials capable of producing a wider range of colors than a bistable material. In particular, we can expect to control the output color by adjusting the length of the helix. In this system, the shape of the high-pitch helix is almost a straight line so the transition between three configurations is analogous to the alignment transition from parallel normally white mode to twisted helical mode found in nematic liquid crystal molecules that ushered in the era of liquid crystal color displays. Moreover, the substantial disparity in energy within a tristable status may be utilized for high-density energy storage.

## Data Availability

The data that support the findings of this study are available from the corresponding author upon reasonable request.

## Appendix: Expressions for static equations and stability criterion

From Eqs. (2)–(4), we can find the explicit forms of static equations as follows,

9 $$\begin{aligned}{} & {} (\cos ^2\psi +k_2\sin ^2\psi )\ddot{\theta }+(k_2-1)\sin 2\psi \dot{\theta }\dot{\psi }+[(1-k_2)\cos 2\psi \sin ^2\theta +c_0(k_2\cos \alpha \cos \psi +\sin \alpha \sin \psi )\nonumber \\{} & {} -k_3\cos 2\theta ]\dot{\psi }+k_3\tau _0\cos 2\theta -[(k_2-k_3+1+(k_2-1)\cos 2\psi )\sin ^2\theta +k_3\cos ^2\theta ]\cos \theta \sin \theta \nonumber \\{} & {} +c_0\sin 2\theta (k_2\cos \alpha \cos \psi +\sin \alpha \sin \psi )=0, \end{aligned}$$

10 $$\begin{aligned}{} & {} 2k_3\ddot{\psi }-(k_2-1)\sin 2\psi \dot{\theta }^2-2[c_0(k_2\cos \alpha \cos \psi +\sin \alpha \sin \psi ) -(k_2-1)\cos 2\psi \sin ^2\theta -k_3\cos 2\theta ]\dot{\theta }\nonumber \\{} & {} +[(k_2-1)\sin ^2\theta \sin 2\psi +2c_0(\sin \alpha \cos \psi -k_2\cos \alpha \sin \psi )]\sin ^2\theta =0. \end{aligned}$$

Equations (9) and (10) are second order nonlinear differential equations but it is straightforward to find their *s*-independent solutions, as given by Eqs. (5)-(6).

Let $\theta =\theta _h+\Delta \theta$, $\psi =\psi _h+\Delta \psi$, where $\theta _h$ and $\psi _h$ are $s-$independent, and keep the terms up to the first order of $\Delta \theta$ and $\Delta \psi$, Eqs. (9) and (10) become

11 $$\begin{aligned} c_{11}\ddot{\Delta \theta }+c_{12}\dot{\Delta \theta }+c_{13}\Delta \theta +c_{14}\ddot{\Delta \psi } +c_{15}\dot{\Delta \psi }+c_{16}\Delta \psi= & {} 0, \end{aligned}$$

12 $$\begin{aligned} c_{21}\ddot{\Delta \theta }+c_{22}\dot{\Delta \theta }+c_{23}\Delta \theta +c_{24}\ddot{\Delta \psi } +c_{25}\dot{\Delta \psi }+c_{26}\Delta \psi= & {} 0, \end{aligned}$$

where $c_{ij}$’s are also $s-$independent and

13 $$\begin{aligned} c_{11}= & {} \cos ^2\psi _h+k_2\sin ^2\psi _h, c_{12}=c_{14}=c_{21}=c_{25}=0, c_{24}=k_3,\end{aligned}$$

14 $$\begin{aligned} c_{13}= & {} -k_3 (\cos ^4\theta _h+\sin ^4\theta _h)- 3\cos ^2\theta _h \sin ^2\theta _h[1+k_2-2k_3+(k_2-1)(2\cos ^2\psi _h-1)]\nonumber \\{} & {} +2\sin ^4\theta _h(k_2\cos ^2\psi _h+\sin ^2\psi _h)+2 c_0\cos 2\theta _h(\sin \alpha \sin \psi _h+ k_2\cos \alpha \cos \psi _h)-2k_3\tau _0\sin 2\theta _h,\end{aligned}$$

15 $$\begin{aligned} c_{15}= & {} k_3(1-2\cos ^2\theta _h)+(k_2-1)(1-2\cos ^2\psi _h)\sin ^2\theta _h+c_0 (k_2\cos \alpha \cos \psi _h+\sin \alpha \sin \psi _h),\end{aligned}$$

16 $$\begin{aligned} c_{16}= & {} c_0 \sin 2\theta _h(\cos \psi _h \sin \alpha -k_2 \cos \alpha \sin \psi _h)+2(k_2-1)\cos \theta _h\sin ^3\theta _h \sin 2\psi _h, \end{aligned}$$

17 $$\begin{aligned} c_{22}= & {} k_3 (2\cos ^2\theta _h-1)-c_0(k_2 \cos \alpha \cos \psi _h+\sin \alpha \sin \psi _h)+(k_2-1) (2\cos ^2\psi _h-1) \sin ^2\theta _h,\end{aligned}$$

18 $$\begin{aligned} c_{23}= & {} \sin 2\theta _h [c_0(\cos \psi _h\sin \alpha -k_2 \cos \alpha \sin \psi _h)+(k_2-1) \sin ^2\theta _h \sin 2\psi _h],\end{aligned}$$

19 $$\begin{aligned} c_{26}= & {} \sin ^2\theta _h [-c_0 (k_2 \cos \alpha \cos \psi _h+\sin \psi _h\sin \alpha )+(k_2-1)\sin ^2\theta _h( \cos ^2\psi _h-\sin ^2\psi _h)]. \end{aligned}$$

Assuming $\Delta \theta =B_\theta e^{\gamma s+\delta }$ and $\Delta \psi =B_\psi e^{\gamma s+\delta }$, where $B_\theta$ and $B_\psi$ are again $s-$independent, Eqs. (11) and (12) become

20 $$\begin{aligned} (c_{11}\gamma ^2+c_{13})B_\theta +(c_{15}\gamma +c_{16})B_\psi =0, \ (c_{22}\gamma +c_{23})B_\theta +(c_{24}\gamma ^2+c_{26})B_\psi =0. \end{aligned}$$

Now demanding $B_\theta \ne 0$ and $B_\psi \ne 0$, we obtain a quadratic equation of $\gamma ^2$, i.e.,

21 $$\begin{aligned} \Delta\equiv & {} (c_{11}\gamma ^2+c_{13})(c_{24}\gamma ^2+c_{26})-(c_{15}\gamma +c_{16}) (c_{22}\gamma +c_{23})\nonumber \\= & {} k_3[k_2+(1-k_2)v^2]\gamma ^4+C_2\gamma ^2+C_0=0,\end{aligned}$$

22 $$\begin{aligned} C_0= & {} c_{13}c_{26}-c_{16}c_{23},\ C_2=c_{11}c_{26}+c_{13}c_{24}-c_{15}c_{22}. \end{aligned}$$

If both $\gamma ^2<0$, then the helix is at least metastable because all $\gamma$’s are imaginary so $\Delta \theta$ and $\Delta \psi$ will remain small at an arbitrary *s*, or a small disturbance on the filament cannot induce a serious deviation from the helical configuration. Otherwise, the helix is unstable since at least one Re($\gamma )>0$ so both $\Delta \theta$ and $\Delta \psi$ will increase rapidly with increasing *s*, and the filament will deviate considerably from a helix at large *s*, even if both $B_\theta$ and $B_\psi$ are small. Therefore, $\gamma ^2$ can serve as a stability criterion to assess the stability of a helix.
